# Supplementary material for: Identification and structural analysis of the tripartite α-pore forming toxin of Aeromonas hydrophila
Source: Nat Commun. 2019 Jul 1;10:2900. doi: 10.1038/s41467-019-10777-x (PMC6602965; doi:10.1038/s41467-019-10777-x)
Supplement: Supplementary file 1 — Supplementary Information [file 41467_2019_10777_MOESM1_ESM.pdf]

Identification and structural analysis of the tripartite α-pore forming toxin of *Aeromonas hydrophila.*

J. Wilson, A. Churchill-Angus, et al.

Supplementary Information

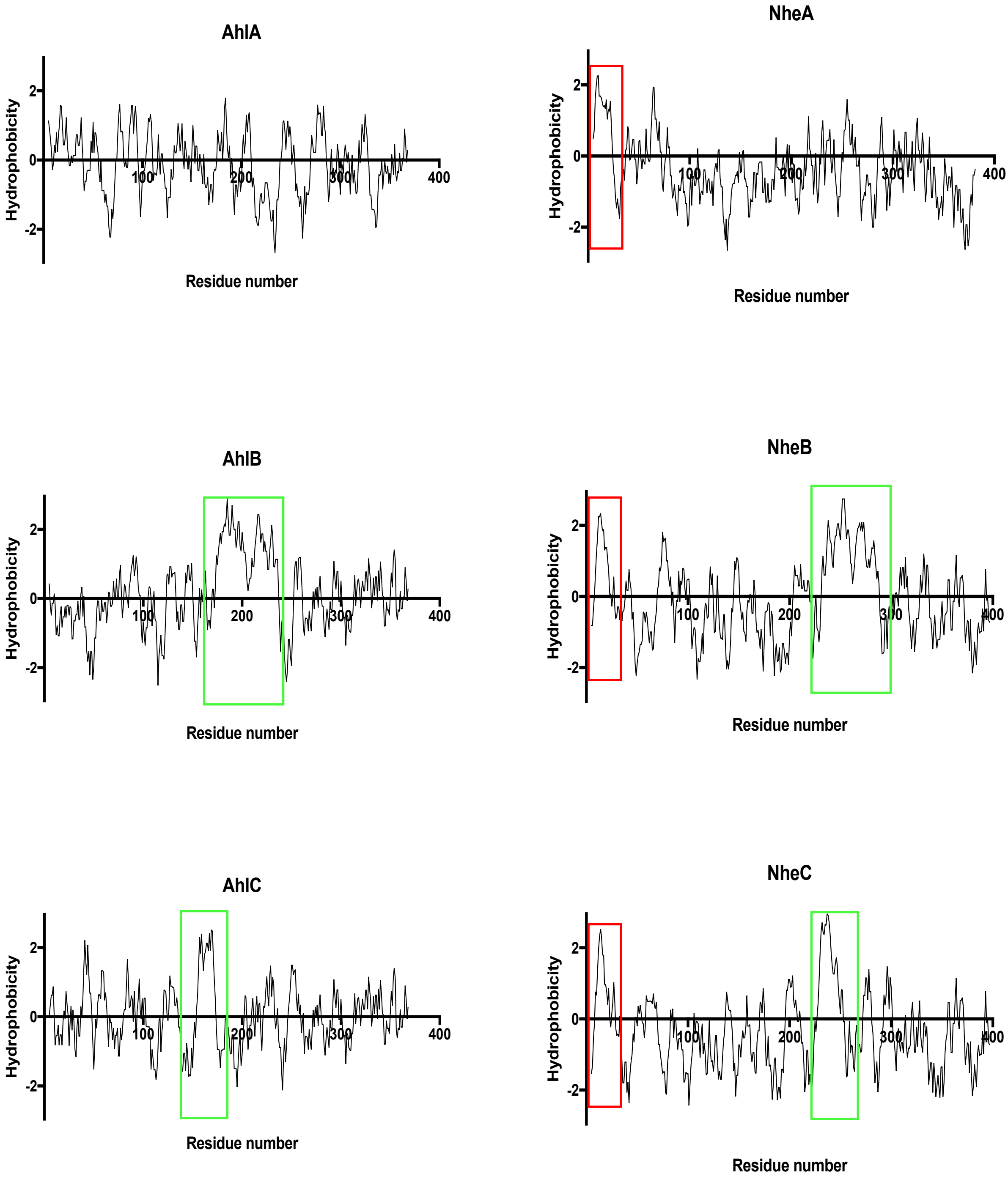

Supplementary Figure 1. Kyte and Doolittle hydrophathy plots. Kyte and Doolittle plots for *B. cereus* NheA, NheB, NheC and *A. hydrophila* AhlA, AhlB, AhlC generated using ProtScale[1] . AhlB, NheB, AhlC and NheC have a single predicted transmembrane region (green box) (>1.5), while AhlA and NheA have no predicted transmembrane region. Red boxes highlight the predicted signal sequences (SignalP [2]) in Gram positive NheA, NheB, NheC proteins

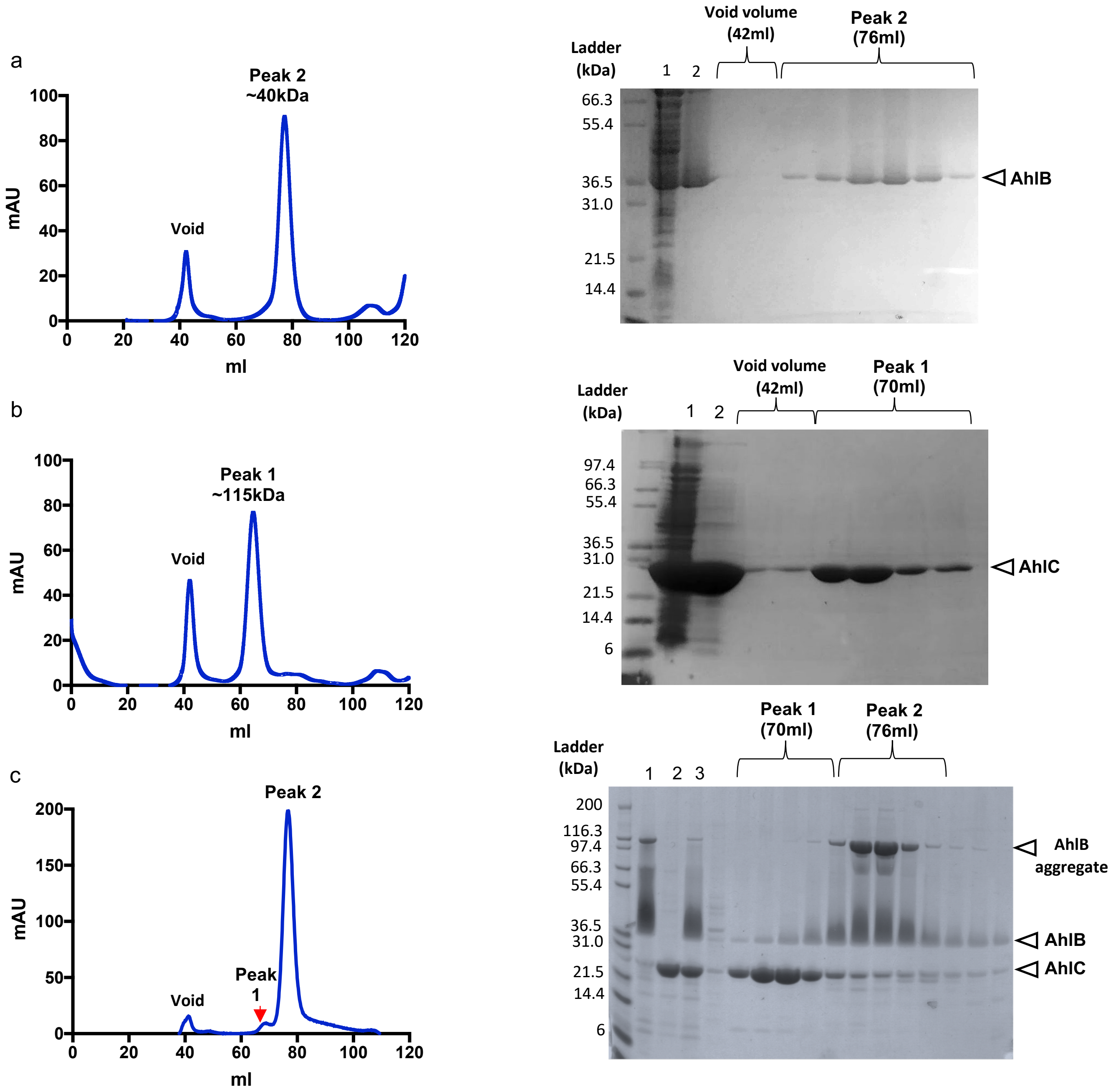

Supplementary Figure 2. Gel filtration purification for AhlB, AhlC, and AhlB+C in solution. Gel filtration UV trace chromatogram (left) and SDS-PAGE (right). (**a**) AhlB forms a monomer in solution. Lane 1 and 2 of the SDS-PAGE show cell free extract (CFE) and Gel filtration load respectively. (**b**) AhlC forms a tetramer in solution. Lane 1 and 2 of the SDS-PAGE show CFE and Gel filtration load (**c**) A 1:2(w/w) mixture of AhlB (peak 2) and AhlC (peak 1) elutes as two separate peaks, showing AhlB and AhlC do not interact in solution. The reduced absorbance of AhlC relative to AhlB because AhlC contains no Trp residues and the extinction coefficients are 1490 M^{-1} cm^{-1} and 25440 M^{-1} cm^{-1} for AhlC and AhlB respectively. Lane 1, 2 and 3 of the SDS-PAGE show AhlB, AhlC, and AhlB/AhlC Gel filtration load respectively. AhlB runs as a partially aggregated (90 kDa) species on SDS-PAGE when boiled[3]. Monomric AhlB is smeared as no stack was used on the gel.

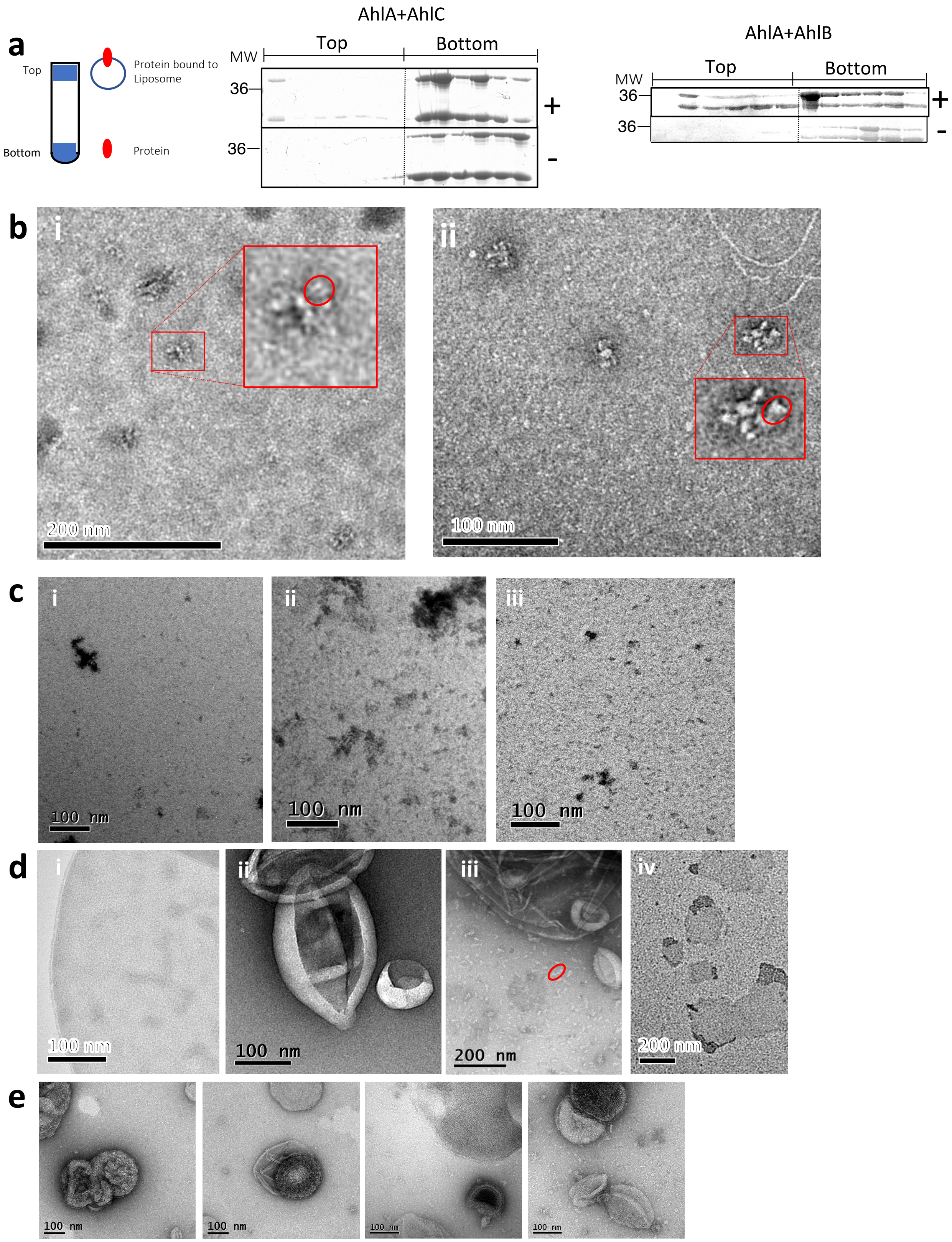

Supplementary Figure 3 EM micrographs of erythrocytes and liposomes. (a) SDS-gels of top and bottom fractions from liposome float assays where AhlA and AhlC, and AhlA and AhlB were incubated with liposomes (+) and without liposomes (-). A schematic of the ultracentrifuge tube shows the location of the top and bottom fractions and in which fractions liposomes and protein are expected. Source data are provided as Source Data file. **(b)** Negative stain EM of ultracentrifugation pellet from AhlA+AhlB+AhlC (i), and AhlB +AhlC (ii). Side views of groups of pores in micelles can be seen in both cases. Enlarged images are highlighted in red with individual pores in red circles. (**c**) Fractions from negative controls of liposome float assays for AhlB (i), AhlBC (ii) and AhlABC (iii). No ordered pore like complexes are seen when these proteins are incubated together in the absence of liposomes. (**d**) Erythrocyte membrane (i) and liposomes (ii) in the absence of AhlABC components. Liposomes incubated with AhlA (iii), and AhlC (iv). AhlA forms extended fibrous aggregates on the surface of the EM grid (highlighted by red oval), but does not form any ordered assemblies on the surfaces of the liposomes. Liposomes are destroyed by the presence of AhlC and form flat sheets on the carbon grid. (**e**) AhlB and AhlC together saturate some liposomes with pores while others are left empty.

a

b

c

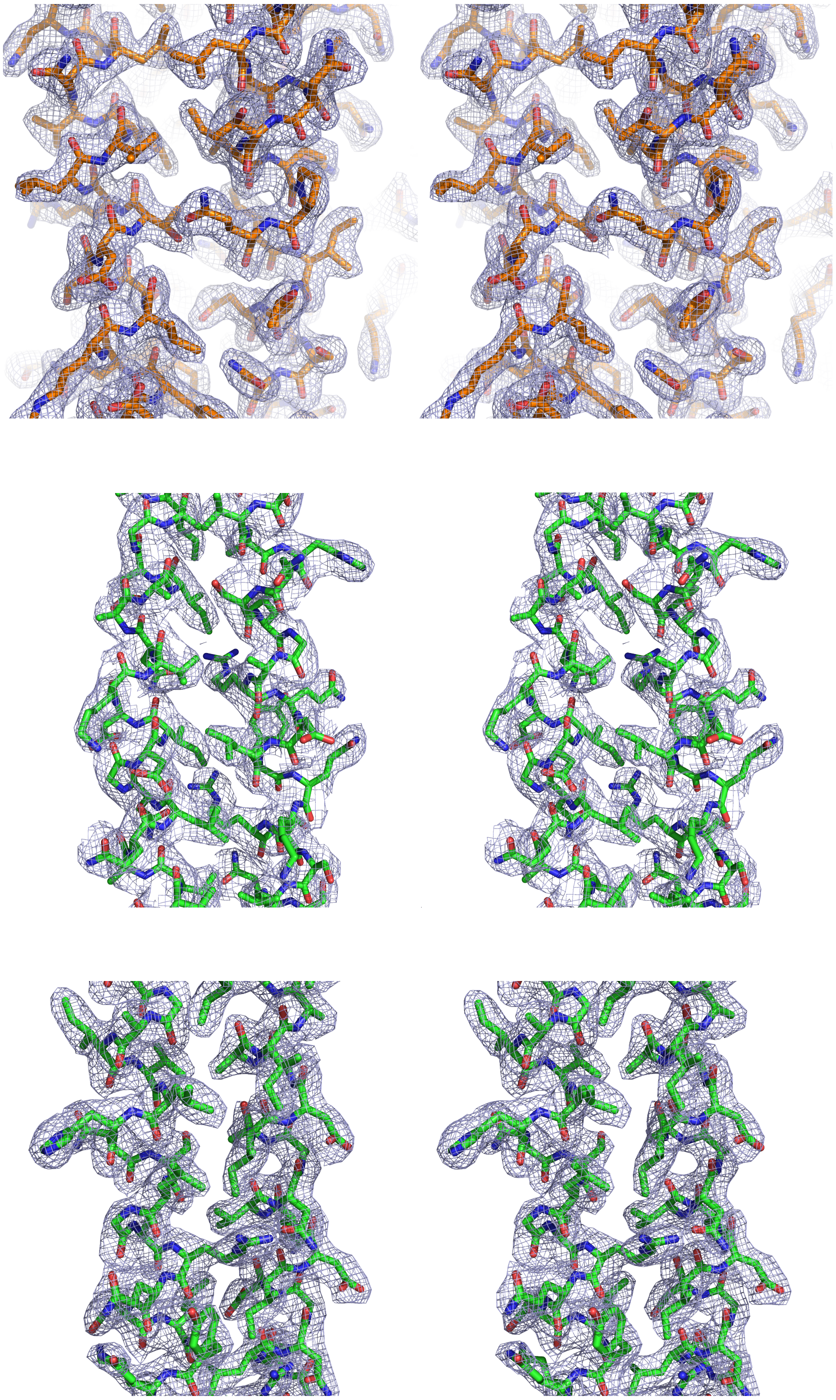

Supplementary Figure 4. Stereo images of areas of the 2Fo-Fc electron density maps of AhlB. An area of the density map (blue mesh), contoured at 1.0 σ, **(a)** AhlB soluble(D63-N79 alpha 2 and Y117-N132 alpha 3) **(b)** SeMet AhlB pore, (V159-I178 alpha 3 and V233-L251 alpha 4) **(c)** AhlB pore Form 2 (Q162-G180 alpha 3 and A229-Q246 alpha 4).

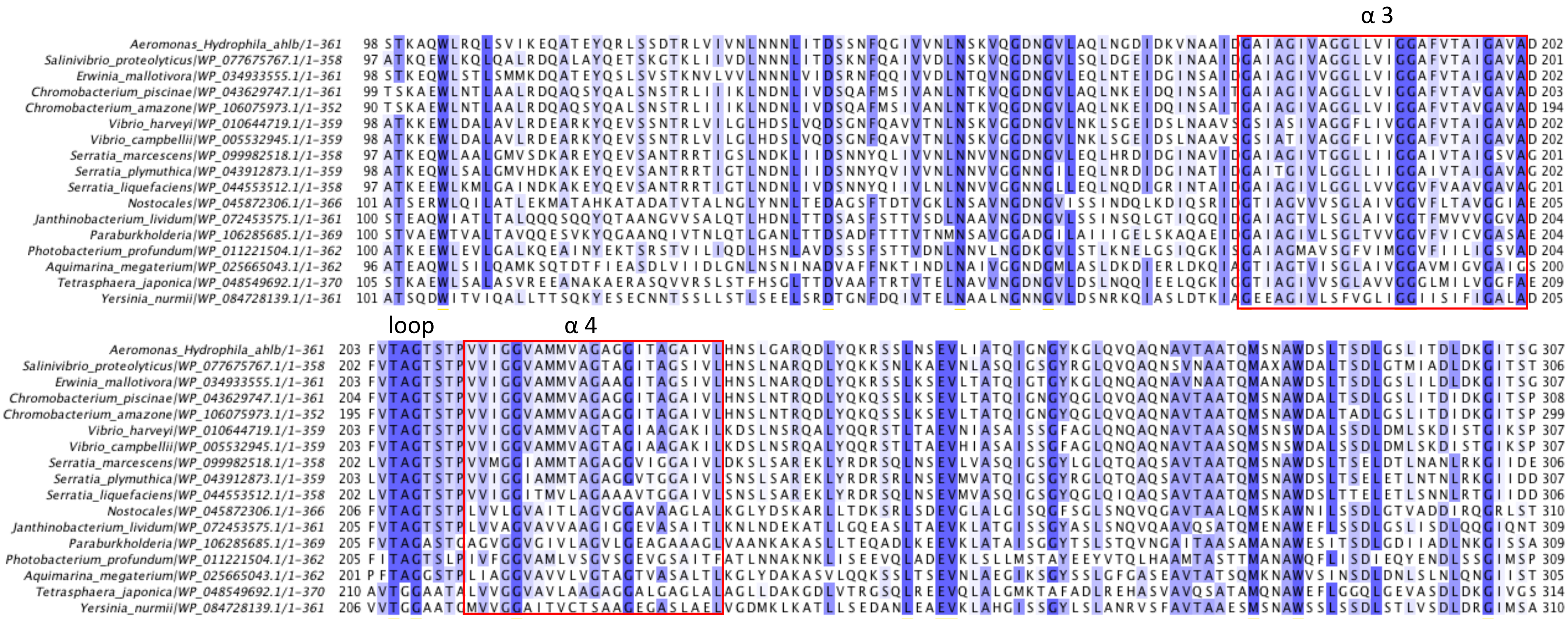

Supplementary Figure 5. Sequence analysis of AhlB. Sequence alignment, using Tcoffee [4], of AhlB against other Gram negative bacteria identified using BlastP [5]. Region G176-A201 (α 3), loop, and V212-L234 (α 4) are highlighted. The two hydrophobic helices are conserved in all bacteria as well as the highly conserved loop region.

a

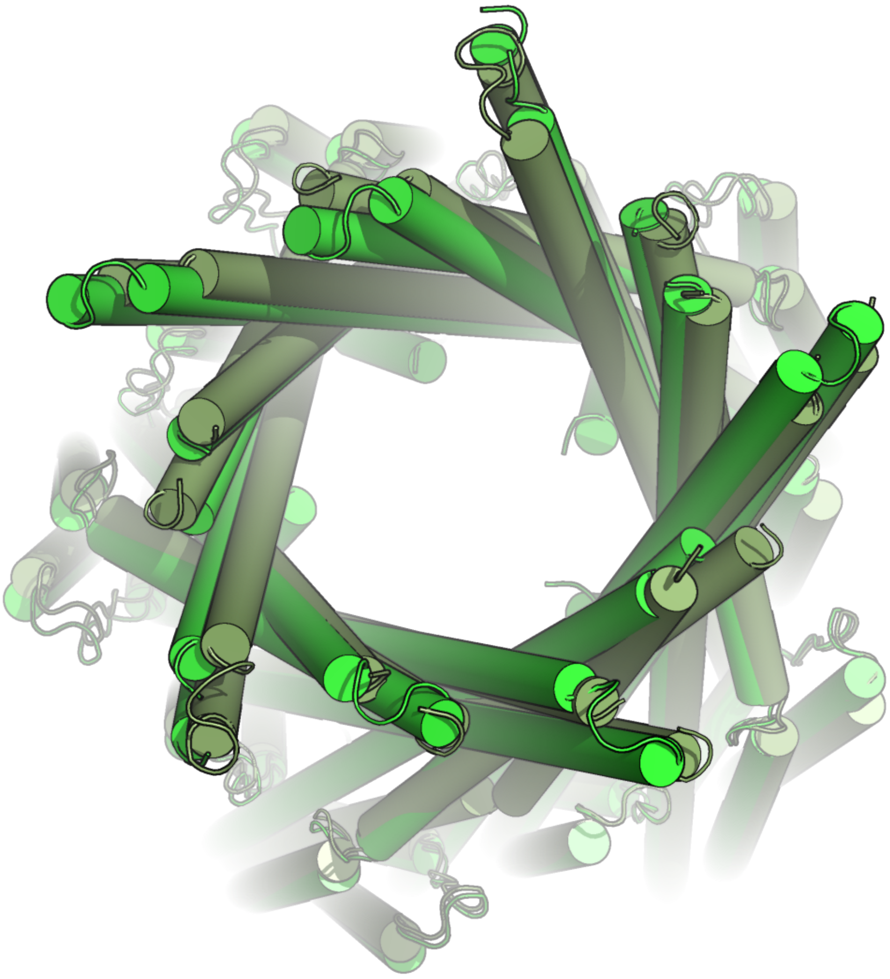

Supplementary Figure 6. Different packing of head domains in each crystal form of AhlB pore shows flexibility in the head domain. (**a**) Superposition of AhlB Form 1 (dark green) and AhlB Form2 (light green) based on residues from the tail domains, viewed from the head showing differences in the positioning of the heads in each form, suggesting flexibility of the AhlB head domain.

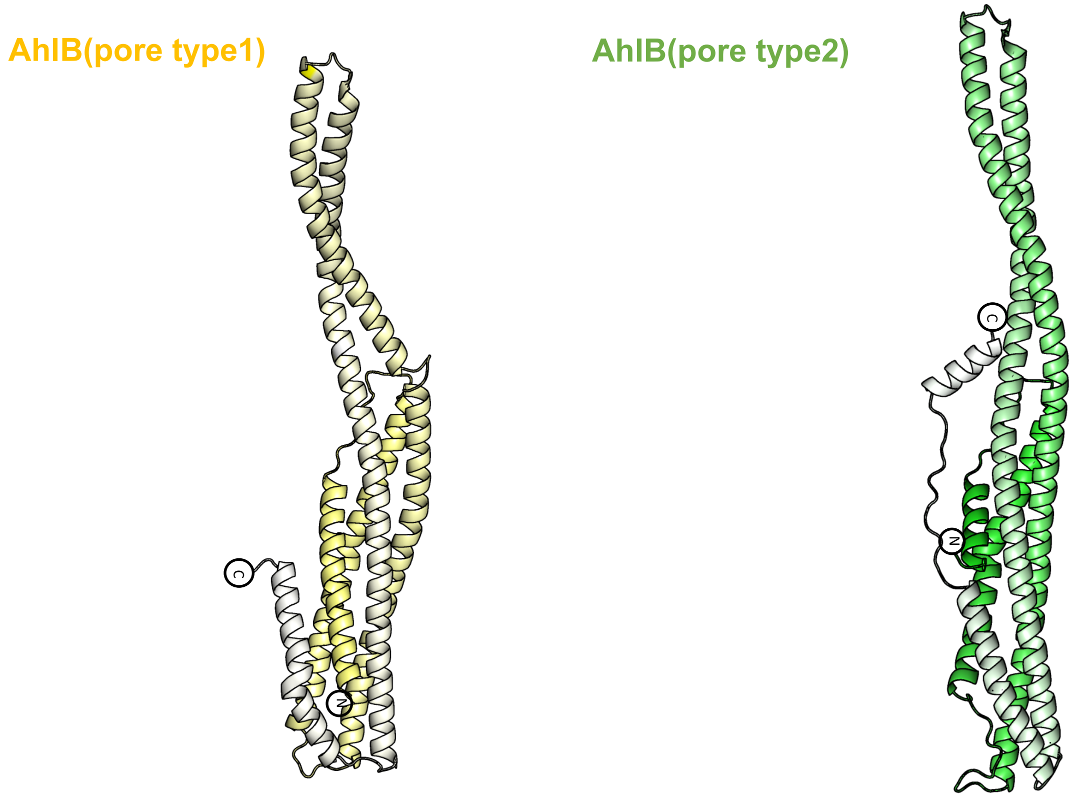

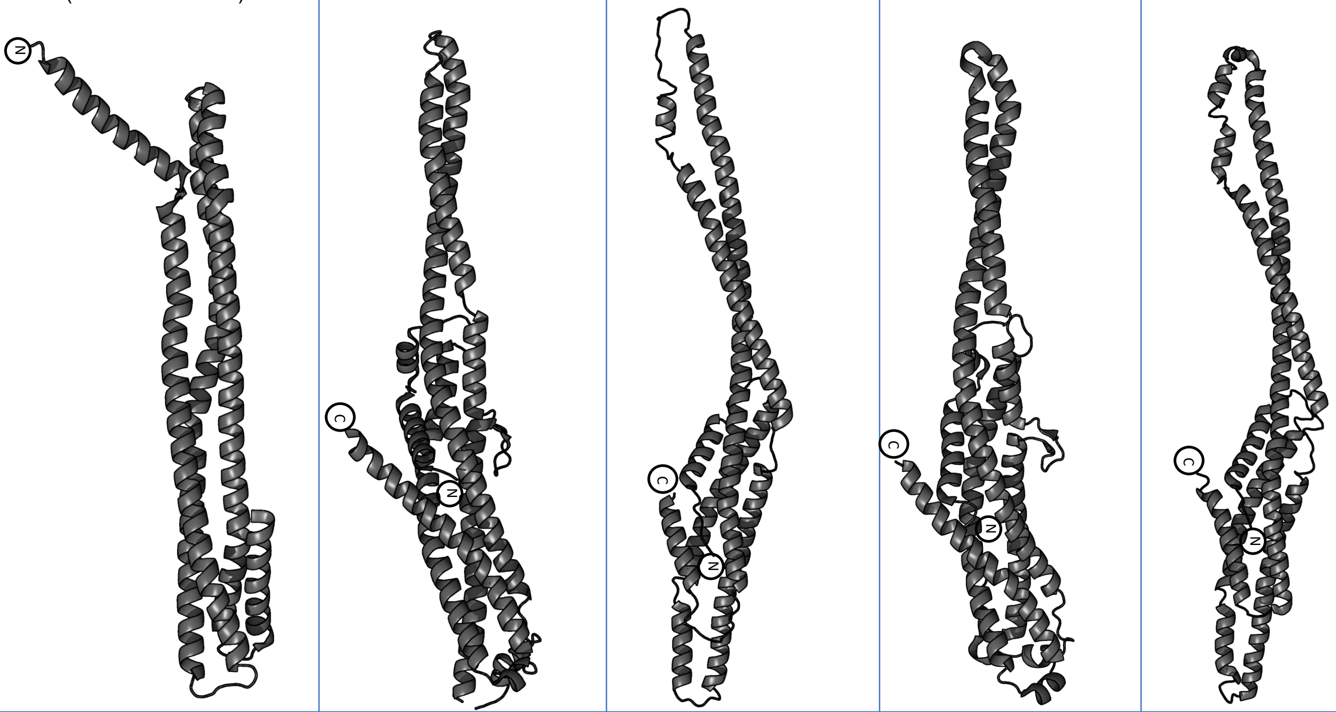

	ClyA(pore) (PDB code 2WCD)	YaxA(pore) (PDB code 6EL1)	YaxB(pore) (PDB code 6EL1)	XaxA(pore) (PDB code 6GY6)	XaxB(pore) (PDB code 6GY6)
**AhlB Type1**	Z-score 10.9<br>RMSD 5.2 Å<br>% ID 7%	Z-score 15.5<br>RMSD 7.5 Å<br>% ID 7%	Z-score 12.7<br>RMSD 4.3 Å<br>% ID 7 %	Z-score 13.5<br>RMSD 4.5Å<br>% ID 8%	Z-score 5.6<br>RMSD 3.8Å<br>% ID 7%
**AhlB Type2**	Z-score 13.7<br>RMSD 4.1 Å<br>% ID 10%	Z-score 18.1<br>RMSD 3.7 Å<br>% ID 9%	Z-score 11.1<br>RMSD 3.5 Å<br>% ID 7%	Z-score 20<br>RMSD 5.6Å<br>% ID 9%	Z-score 12.4<br>RMSD 3.5Å<br>% ID 7%

Supplementary Figure 7. Structural superposition of AhlB (pore) with ClyA α-PFT family toxins. Superposition of AhlB Type1 (yellow) and AhlB Type2 (green) with ClyA family α-PFT's (grey) (aligned by the DALI server [6]), with Z-scores, RMSD(Cα) and percentage sequence identity (%ID).

a

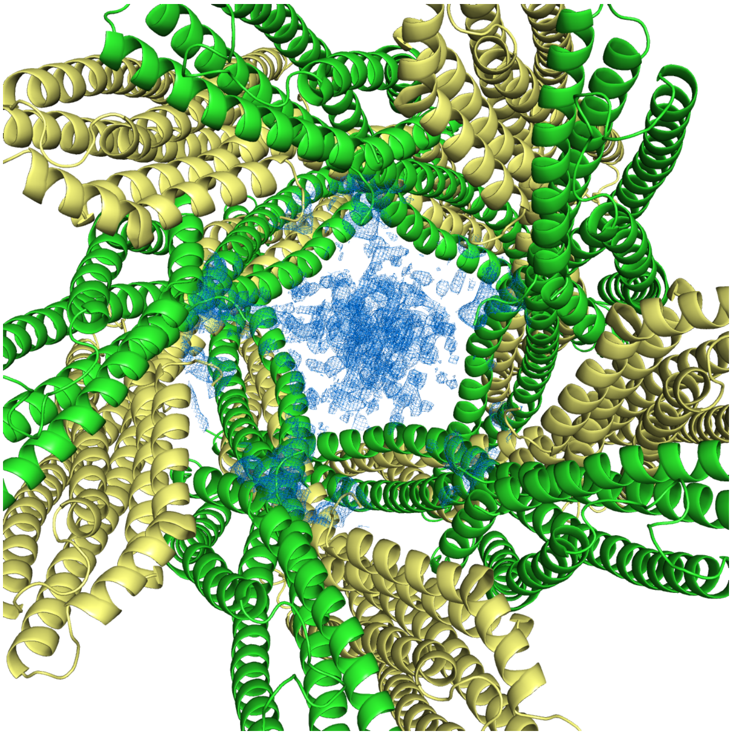

b

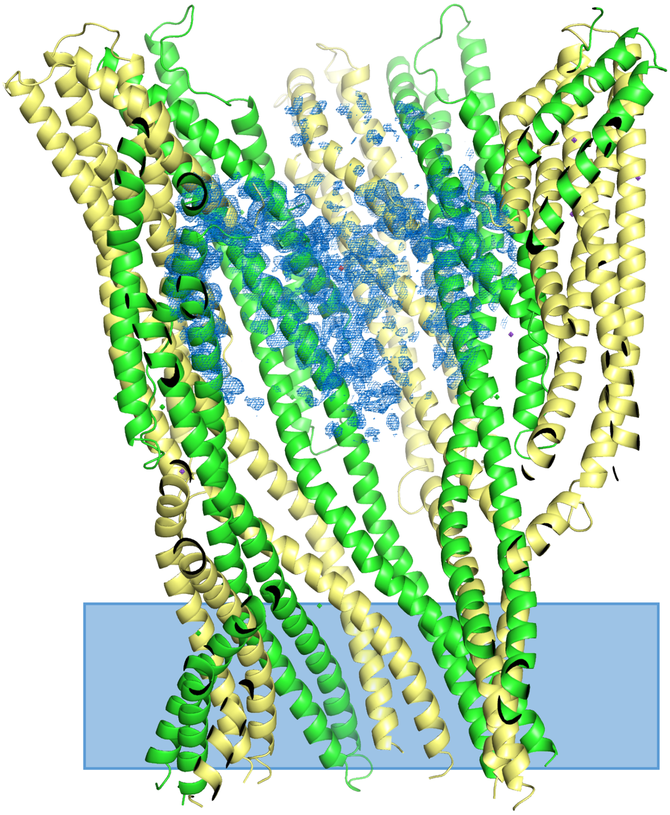

Supplementary Figure 8. Undefined density at the center of the AhlB pore. a) View looking down from the tail of the AhlB pore (green and yellow cartoon helices). 2Fo-Fc Density map, contorted at 0.9 σ (blue mesh), shows a large region of undefined density blocking the center of the AhlB pre-pore. b) side view of the AhlB pore slabbed to show the undefined density at the center. 2Fo-Fc Density map, contorted at 0.9 σ (blue mesh). The membrane is shown as a blue box.

a

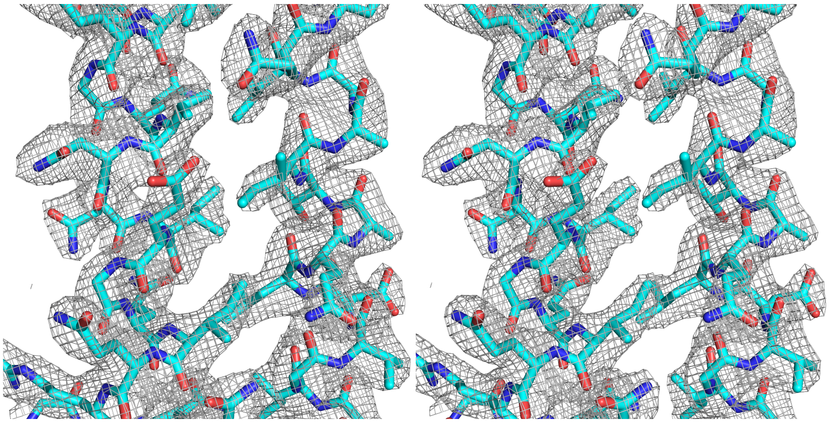

b

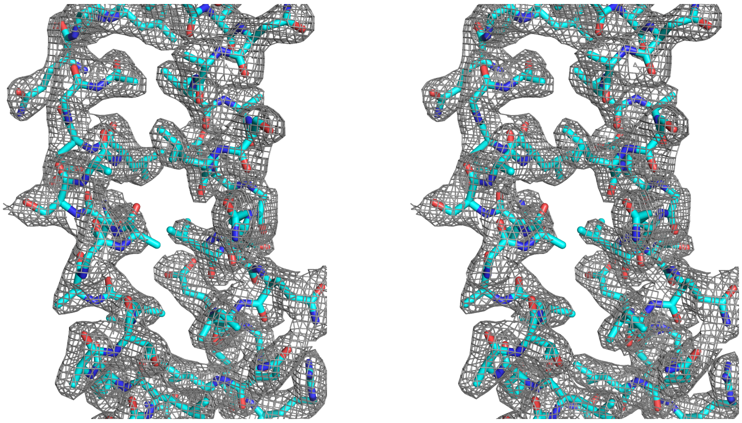

Supplementary Figure 9. Stereo images of an area of the 2Fo-Fc electron density maps of AhlC. An area of the density map (blue mesh), contoured at 1.0 σ, around alpha helices 3 and 4 of chain P from **(a)** AhlC Form 1, (G184-N197 alpha3 and Q127-Q135 alpha4) **(b)** AhlC Form 2 (L127-L145 alpha 3 and G177-R196 alpha 4)

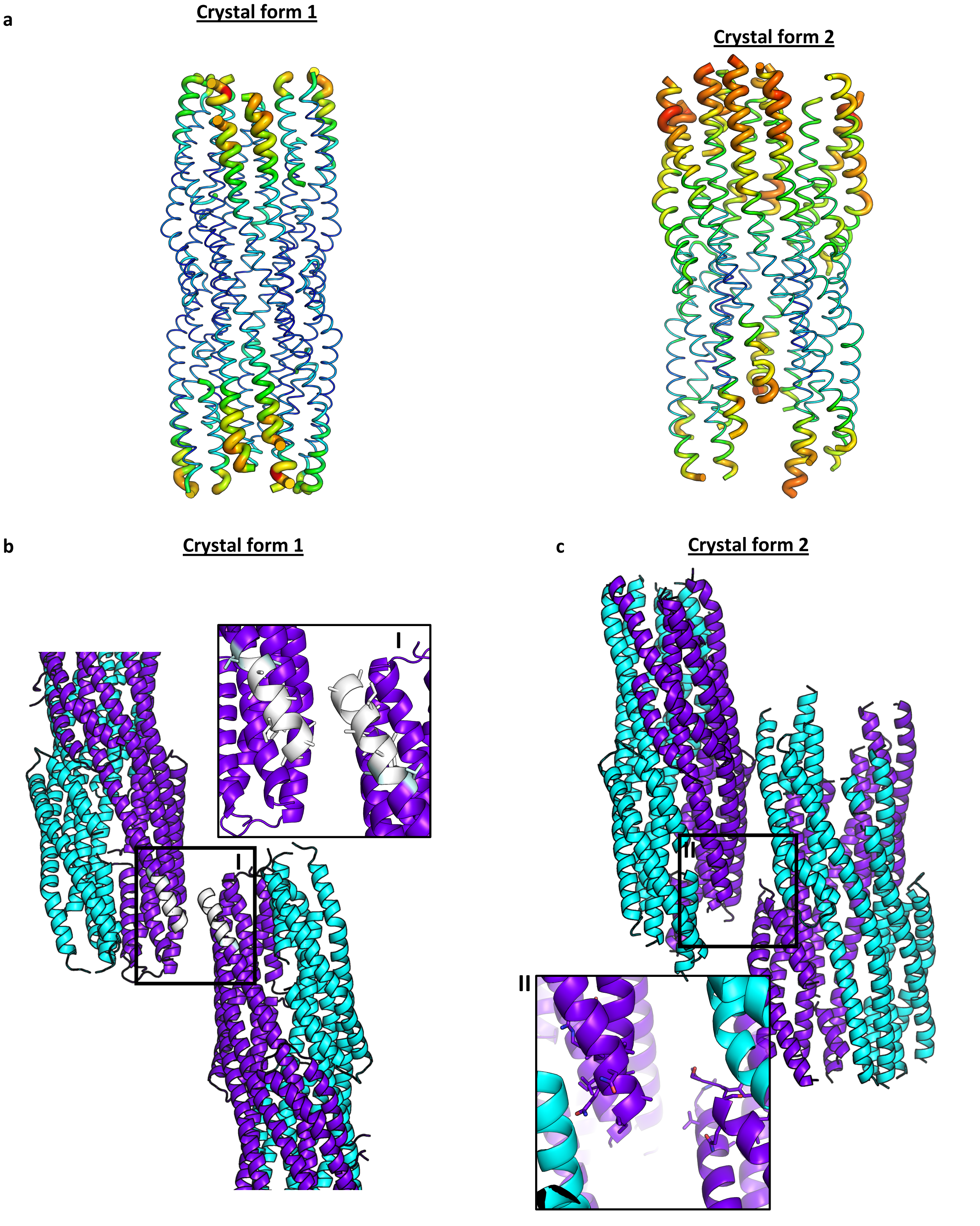

Supplementary Figure 10. Crystal packing of crystal Form 1 and crystal form 2 of AhlC shows flexibility in the head of AhlC. (a) B-factor putty diagram of crystal Form 1 and 2, low B-factor (blue) to high B-factor (red). Crystal form 1 has lower B-factors in the head domain and a more complete structure. Crystal form 2 has higher B-factors in the head domain and is incomplete showing high flexibility. Cartoon diagrams of the crystal packing of AhlC Crystal form 1 (**b**) and Crystal form 2 (**c**). The hydrophobic head (white) of Q and Q' (purple) in Crystal form 1 pack against neighboring hydrophobic heads in symmetry related molecules (**I**). In Crystal form 2 packing prevents hydrophobic interactions and results in disordered heads in Q and S (**II**).

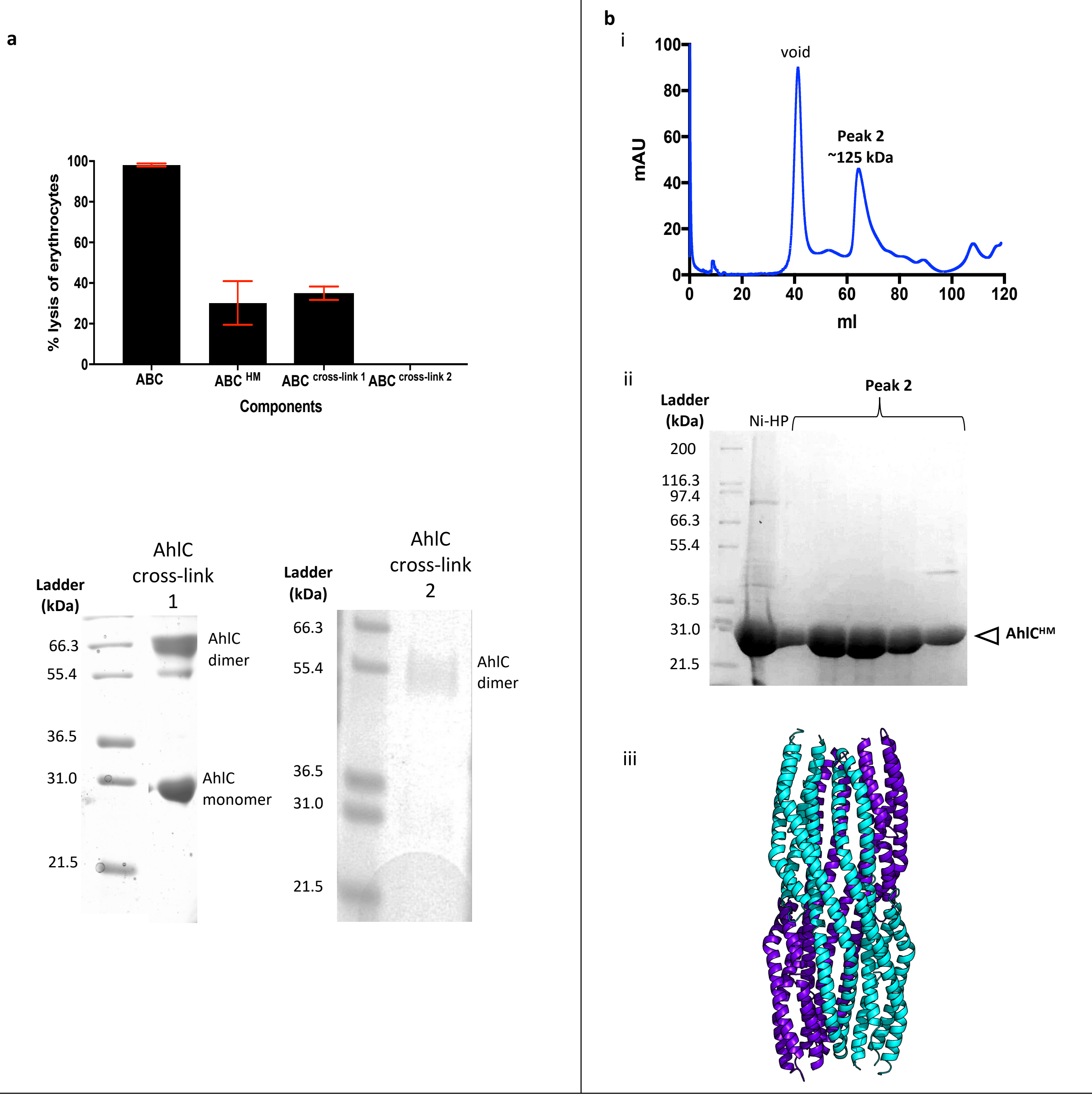

Supplementary Figure 11. AhlC head mutant L156T, L160T, L161T (AhlC^{HM}) purification and haemolytic assays. (**a**) 1μM AhlA and AhlB with AhlC, AhlC^{HM} , AhlC $^{\text{cross-link 1}}$ or AhlC $^{\text{cross-link 2}}$, were added to 0.25% (w/v) horse erythrocytes and incubated for 1hr at 37°C. Percentage haemolysis of 0.25% (w/v) horse erythrocytes by the AHL α-PFT was calculated by measuring absorbance of the supernatant at 542nm, all assays were done in triplicate. Below are SDS-Page gels of cross linked AhlC product in cross-link1 (glutaraldehyde) and cross-link2 (glutaraldehyde + 1-ethyl-3-(3-dimethylaminopropyl)carbodiimide hydrochloride). All assays were carried out in triplicate (n=3) with values corresponding to the mean ± standard deviation. (**b**) Gel filtration UV trace chromatogram (i) and SDS-PAGE (ii),AhlC^{HM} forms a tetramer in solution and in the crystal structure (iii). Source data are provided as Source Data file.

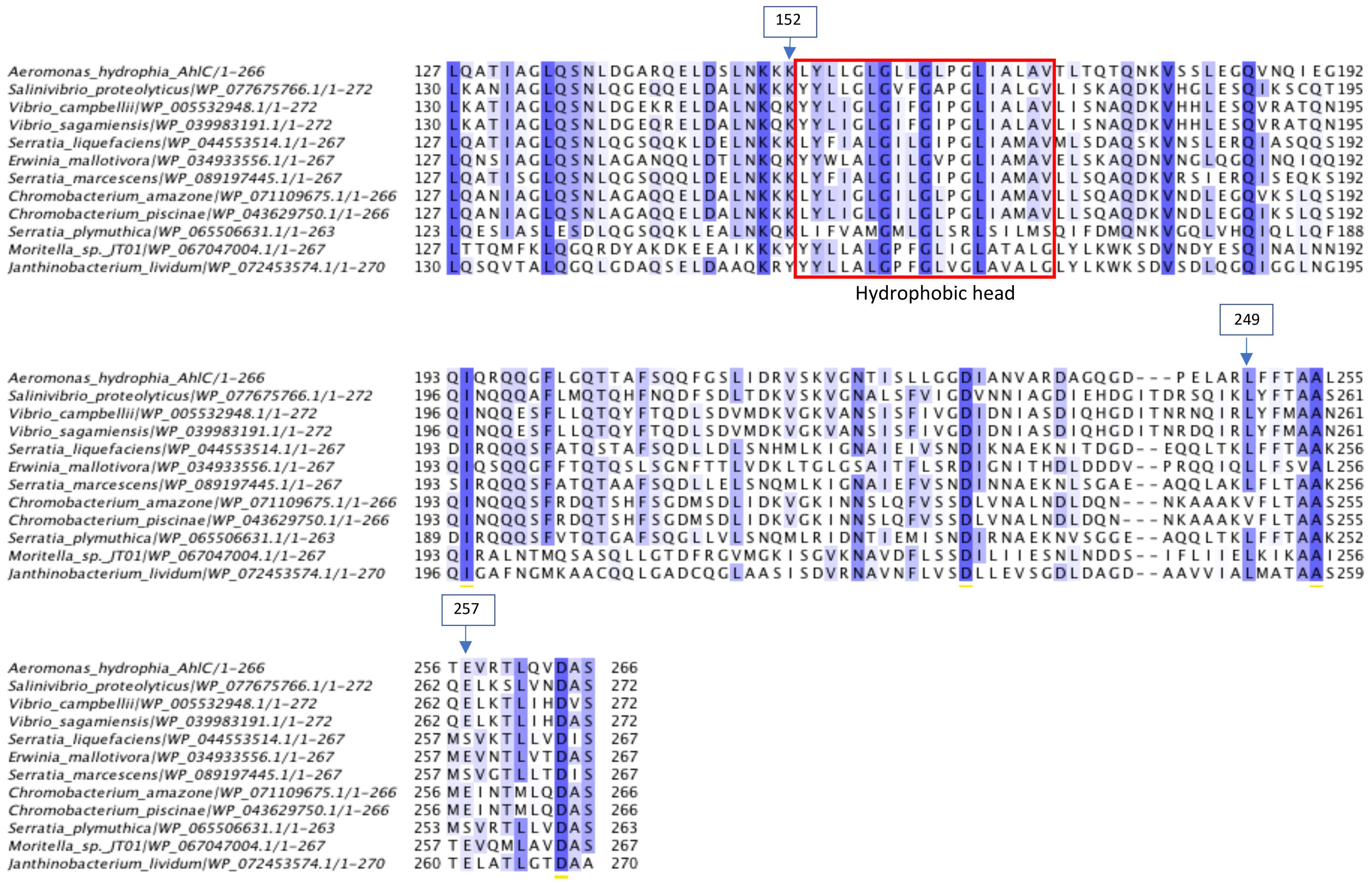

Supplementary Figure 12. Sequence analysis of AhlC. Sequence alignment, using Tcoffee [4], of AhlC against orthologous proteins from other Gram negative bacteria, identified using BlastP [5]. The hydrophobic head and residues K152, L249 and E257 in *A. hydrophila* AhlC are highlighted.

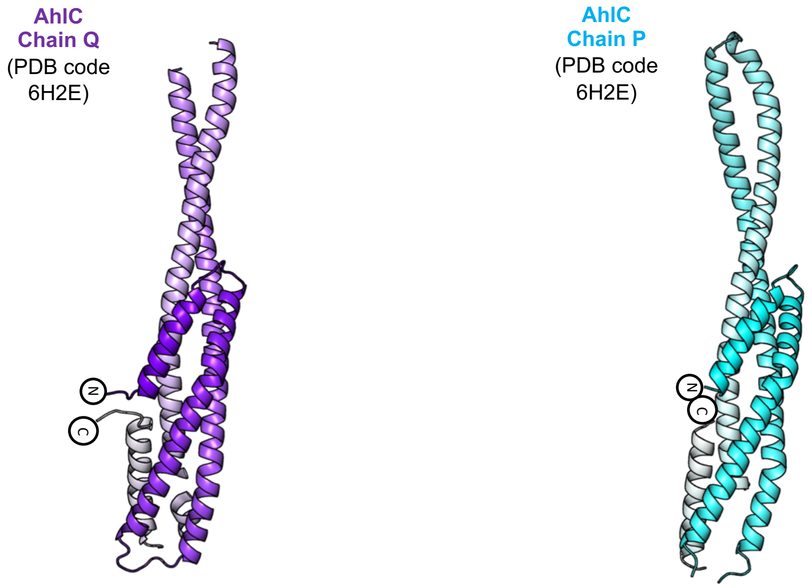

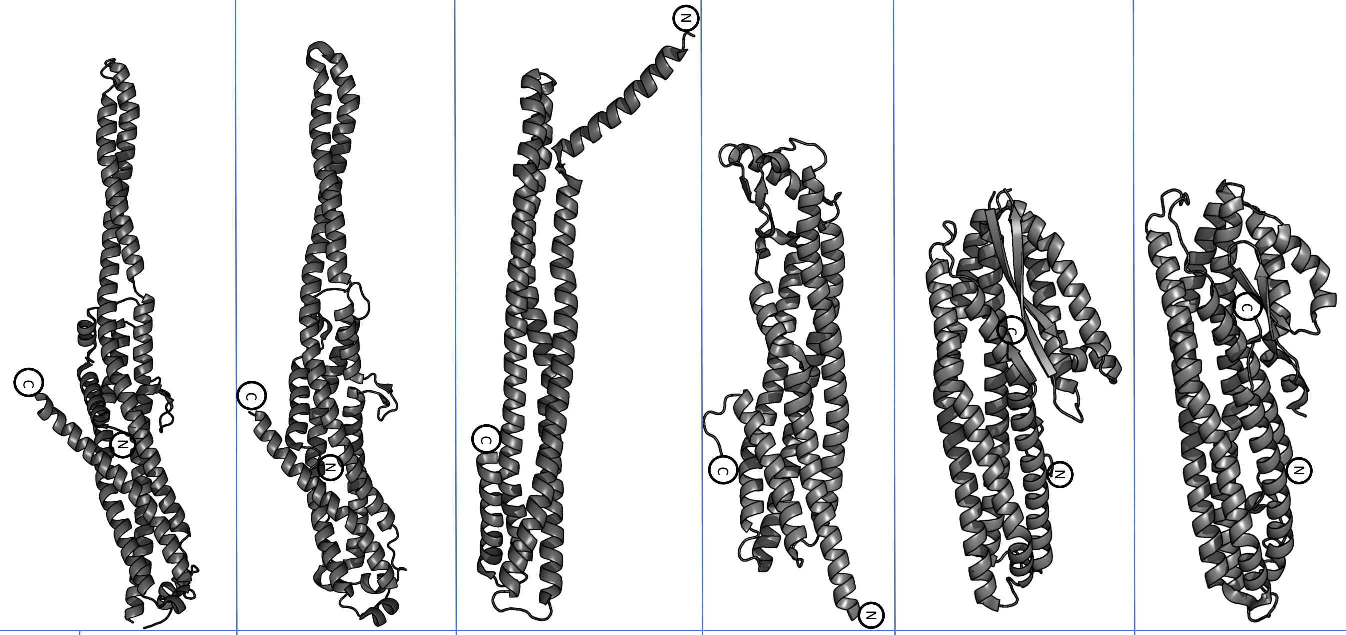

	YaxA (sol) (PDB code 6EK7)	XaxA (sol) (PDB code 6GY8)	ClyA(pore) (PDB code 2WCD)	ClyA (sol) (PDB code 1QOY)	NheA (sol) (PDB code 4K1P)	Hbl-B (sol) (PDB code 2NRJ)
**AhlC Chain Q**	Z-score 17.7<br>RMSD 2.8 Å<br>% ID 12 %	Z-score 18.3<br>RMSD 2.7 Å<br>% ID 11 %	Z-score 13<br>RMSD 3.6 Å<br>% ID 8%	Z-score 9<br>RMSD 7.5 Å<br>% ID 8 %	Z-score 14.2<br>RMSD 3.0 Å<br>% ID 13 %	Z-score 14.3<br>RMSD 3.8 Å<br>% ID 12%
**AhlC Chain P**	Z-score 13.4<br>RMSD 4.0 Å<br>% ID 5 %	Z-score 17<br>RMSD 2.8 Å<br>% ID 11 %	Z-score 13.2<br>RMSD 4.9 Å<br>% ID 7%	Z-score 9<br>RMSD 5 Å<br>% ID 10 %	Z-score 13.2<br>RMSD 2.9 Å<br>% ID 13 %	Z-score 13.4<br>RMSD 3.5 Å<br>% ID 13%

Supplementary Figure 13. Structural superposition of AhlC with ClyA α-PFT family toxins. Superposition of AhlC chain Q (purple) and AhlC chain P (blue) with ClyA family α-PFT's (grey) (aligned by the DALI server[6]), with Z-scores, RMSD(Cα) and percentage sequence identity (%ID).

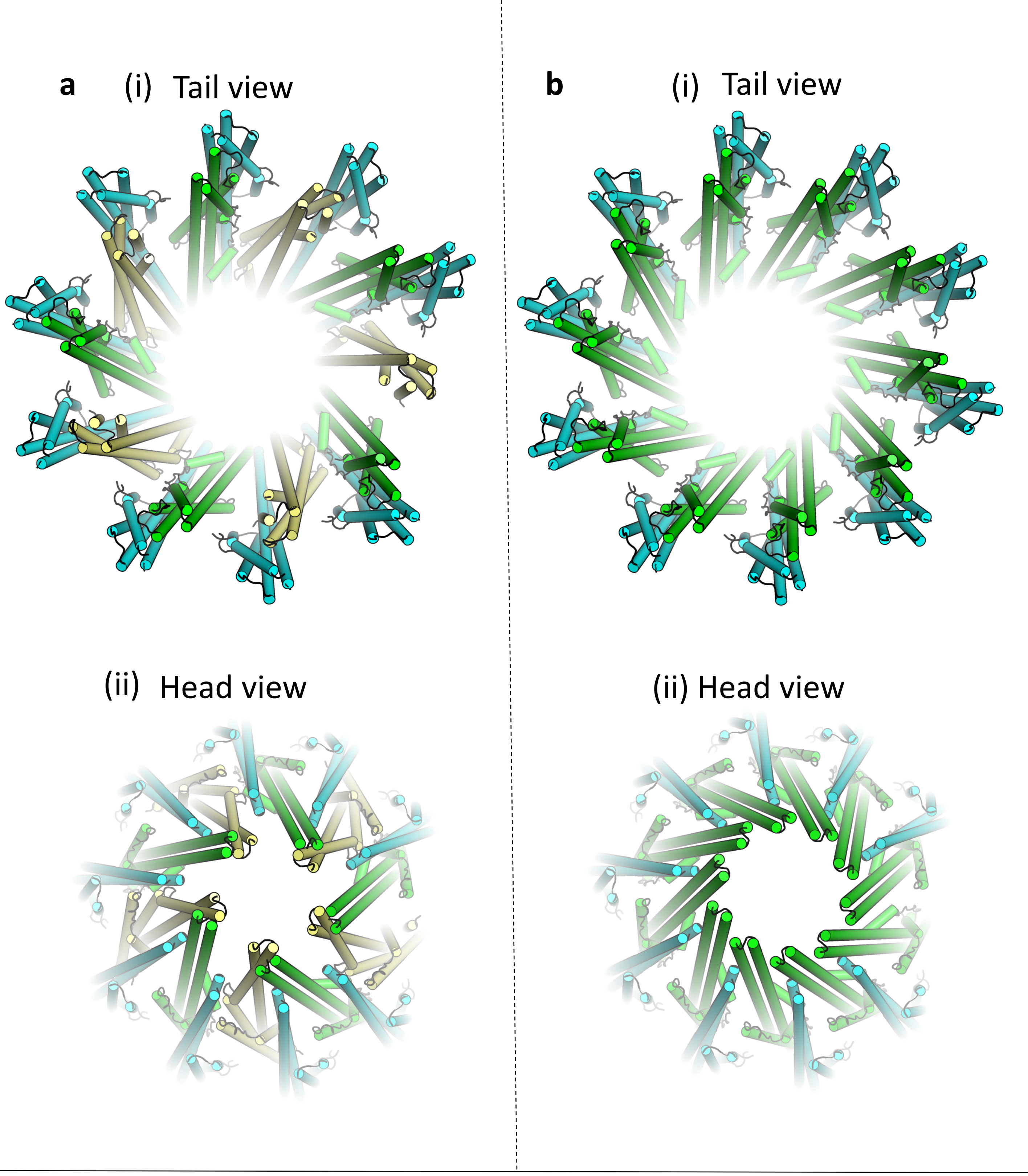

Supplementary Figure 14. Modelling of AhlBC pore into YaxAB
(**a**) Alternating AhlB type1 (yellow) and type2 (green) were aligned with YaxB in the YaxAB pore, while AhlC (cyan) was aligned with YaxA. Clashes can be seen between AhlB type1 and AhlB type2 in both the tail (i) and the head domains (ii). (**b**) AhlB type2 aligned with YaxB to produce a homo-oligomeric AhlB pore, with AhlC aligned with YaxA. In this pore both the tails (i) and heads (ii) of AhlB pack without clashes.

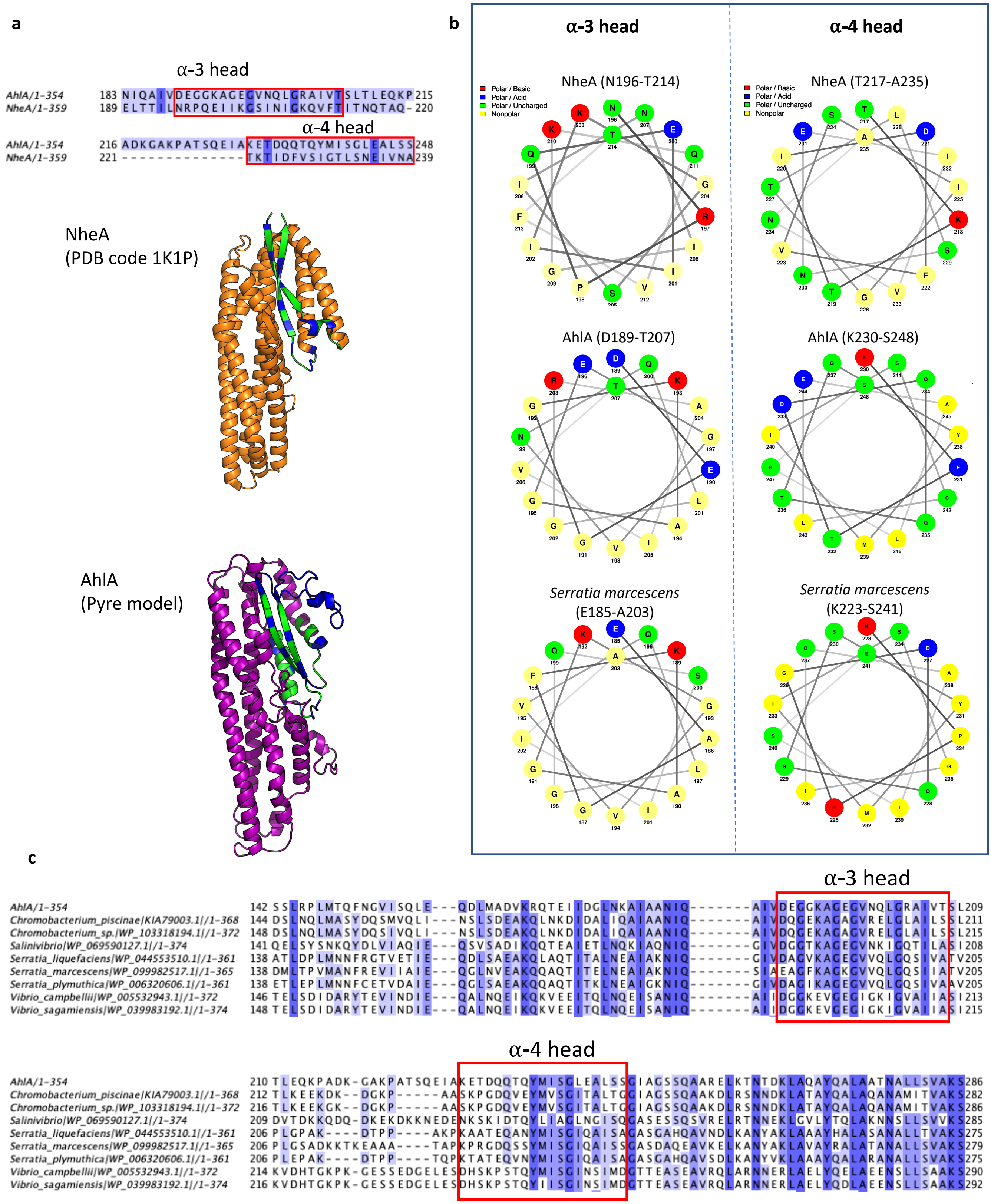

Supplementary Figure 15. Sequence analysis of AhlA. (**a**) Alignment of NheA and AhlA β-tongues which are predicted to construct the α-3 and α-4 heads in the pore forms (upper), with Phyre2 [7] model of AhlA (pink, lower) compared to crystal structure of NheA (orange), both contain β-tongues (green hydrophobic, blue hydrophilic), which could form 2 extended amphipathic helices assuming a similar mechanism of unfolding to that seen in AhlB. (**b**) Helical wheels of predicted transmembrane region of the α-3 and α-4 heads in pore forms of NheA (PDB code 1K1P), AhlA, and *S. marcesecens* generated using NetWheels (Mól, A.R., Castro, M.S. and Fontes, W. NetWheels Tool. http://lbqp.unb.br/NetWheels/). In all three species the α-3 head has an amphipathic helix whith α-4 heads hydrophilic. (**c**) Sequence alignment, using Tcoffee[4], of AhlA against other Gram negative bacteria identified using BlastP [5]. Region D189-T207 (α-3 head) and K230-S248 (α-4 head) are highlighted.

Supplementary Table 1| Gram negative Bacteria identified from BlastP[5] searches as containing full tripartite PFT systems			
**Organism**	**AhlA homologue accession no. and sequence identity (sequence aligned)**	**AhlB homologue accession no. and sequence identity (sequence aligned)**	**AhlC homologue accession no. and sequence identity (sequence aligned)**
Salinovibrio sp.	WP_069590127.1 43% (over 99%)	WP_069590129.1 77% (over 98%)	WP_069590131.1 51% (over 99%)
Erwinia mallotivora	WP_034933552.1 46% (over 99%)	WP_034933555.1 79% (over 99%)	WP_034933556.1 52% (over 100%)
Chromobacterium piscinea	WP_052247043.1 50% (over 98%)	WP_043629747.1 78% (over 97%)	WP_043629750.1 47% (over 100%)
Vibrio campbelli	WP_005532943.1 35% (over 98%)	WP_005532945.1 62% (over 99%)	WP_00532948.1 48% (over 99%)
Serratia marcescens	WP_099982517.1 44% (over 99%)	WP_09982518.1 62% (over 99%)	WP_09982519.1 43% (over 98%)
Serratia plymuthica	WP_006320606.1 43% (over 99%)	WP_043912873.1 62% (over 99%)	WP_06550663.1 38% (over 98%)
Serratia liquefaciens	WP_044553510.1 45% (over 99%)	WP_044553512.1 62% (over 99%)	WP_044553514.1 46% (over 100%)

Supplementary Table 2|Primers used for cloning (restriction sites underlined)	
**Primer**	**Primer sequence**
AhlA his forward	GGC GCT AGG TAC TAC ATA TGA CCA CGA TCG CCA CCC TGG
AhlA his reverse	ATC TAA AGC TTT TAG TGG TGG TGG TGG TGG TGA GCG TCT GGC AGG ATG CC
AhlA link his forward	GGC GGA CAC CAC CAC CAC CAC CAC
AhlA link his reverse	TCC GCC AGC GTC TGG CAG GAT GCC
AhlB his forward	CAG CCC ATA TGA CCA ACG CAA CAA CCA TCA CCA TGG ACC AG
AhlB his reverse	ATC TCG AGG GCG GCC AGG CGC G
AhlC his forward	GGC GCT AGG TAC TAC ATA TGA GCA ACG GCA TTC TTT CC
AhlC his reverse	TAA TCC TCG AGT TAG TGG TGG TGG TGG TGG GAA GCG TCC ACC TGC
AhlC HM SDM forward	GGAACTACTGGGCTGCCGGGCCTCATC
AhlC HM SDM reverse	CAGCCCAGTCAGGTAGAGTTTTTTCTTGTTGAGGGAGTCG

Supplementary references

1. Gasteiger, E. *et al.* Protein Identification and Analysis Tools on the ExPASy Server. *The Proteomics Protocols Handbook* 571–607 (2005).
2. Nielsen, H. Predicting Secretory Proteins with SignalP. *Methods in molecular biology (Clifton, N.J.)* **1611,** 59–73 (2017).
3. Rath, A., Glibowicka, M., Nadeau, V. G., Chen, G. & Deber, C. M. Detergent binding explains anomalous SDS-PAGE migration of membrane proteins. *Proc. Natl. Acad. Sci.* **106,** 1760–1765 (2009).
4. Notredame, C., Higgins, D. G. & Heringa, J. T-coffee: A novel method for fast and accurate multiple sequence alignment. *J. Mol. Biol.* **302,** 205–217 (2000).
5. Altschul, S. F., Gish, W., Miller, W., Myers, E. W. & Lipman, D. J. Basic local alignment search tool. *J. Mol. Biol.* **215,** 403–10 (1990).
6. Holm, L. & Laakso, L. M. Dali server update. *Nucleic Acids Res.* **44,** 351–355 (2016).
7. Kelley, L. A., Mezulis, S., Yates, C. M., Wass, M. N. & Sternberg, M. J. E. The Phyre2 web portal for protein modeling, prediction and analysis. *Nat. Protoc.* **10,** 845–858 (2015).
8. Schrödinger, LLC. *The {PyMOL} Molecular Graphics System, Version~1.8*. (2015).
9. Kleywegt, G.J. Use of non-crystallographic symmetry in protein structure refinement. *Acta Cryst D.* **52,** 842-857. (1996).
