# Supplementary material for: Identification and structural analysis of the tripartite α-pore forming toxin of Aeromonas hydrophila
Source: Nat Commun. 2019 Jul 1;10:2900. doi: 10.1038/s41467-019-10777-x (PMC6602965; doi:10.1038/s41467-019-10777-x)
Supplement: Supplementary file 3 — Description of Additional Supplementary Files [file 41467_2019_10777_MOESM3_ESM.pdf]

Description of Additional Supplementary Files

File name: Supplementary Data 1

Description: BlastP results for AhlA, AhlB, and AhlC with E value<0.01, Aeromonas Taxid excluded. ClyA was not identified by any of the BLAST searches due to very low sequence similarity to any of the Ahl components (E values of 3.7, 0.8 and 2.2 against AhlA, AhlB, AhlC, respectively).

File name: Supplementary Movie 1

Description: A Bi-fold hinge action is required for the conformational change from soluble AhlB to Pore form AhlB. Soluble AhlB (starting image) undergoes a large-scale rearrangement about two hinges to form the Type 1 AhlB pore form. To illustrate this, the soluble AhlB structure (start of movie) was subjected to two rotations of 135º about axes defined through the alpha carbons of 155-250, to form intermediate 1, and 189-224 to form intermediate 2. This structure was then morphed into the AhlB type 1 crystal structure to form intermediate 3 (pause 1), and then morphed again into the AhlB type 2 structure (end of movie), during which α-3 and α4 untwist slightly, and the N and C terminal helices rearrange. The hydrophobic head of AhlB is coloured green, with the loop that protrudes from the distal side of the membrane coloured blue/green. Intermediate coordinate files were produced using Pymol [8] and LSQman [9].
